# Shiftworkers’ attitude to their work hours, positive or negative, and why?

**DOI:** 10.1007/s00420-022-01831-1

**Published:** 2022-02-10

**Authors:** Torbjörn Åkerstedt, Mikael Sallinen, Göran Kecklund

**Affiliations:** 1 Department of Clinical Neuroscience, Karolinska Institute, 17771 Stockholm, Sweden; 2 Department of Psychology, Stress Research Institute, Stockholm University, 10691 Stockholm, Sweden; 3 Finnish Institute of Occupational Health, Work Ability and Working Careers, 00250 Helsinki, Finland

**Keywords:** Shift work, Night work, Daily rest, Split duty, Health

## Abstract

**Objective:**

Shift work is associated with impaired health and safety but there is a lack of systematic knowledge of shift workers attitude to their shift systems. This may be important for the ability to retain valuable personnel in the company/organization, and to attract new employees. The purpose of the present study was to investigate: the prevalence of shift characteristics (nights, long shifts, short rest, etc.) in traditional shift systems, the workers’ attitude to their shift systems, if combinations of problematic shift characteristics are associated with the workers’ attitude, and if work stress and poor sleep, fatigue, or social difficulties are associated with attitudes to shift systems.

**Methods:**

A representative sample of 3,500 individuals with non-day work in the general population of Sweden were asked to participate in the study. A total of 1965 workers remained after drop-outs. The material was analyzed by Chi2 analysis and hierarchical multiple regression.

**Results:**

The results showed that traditional shift systems included many more shift characteristics than those constituting the core of the systems. All included day work, for example. 90.2% of those with roster work had shifts > 10 h at least once a month. 66.9% of those with roster work without nights had < 11 h rest between shifts at least once a month. Less than 25% of the respondents had a rather or very negative attitude to their shift system, with the lowest level for those who work either fixed days or nights (7.6 and 5.7%, respectively) and highest for three-shift work (21.2%) and roster work without night work (24.4%). Shiftwork or roster work with nights had highest levels (> 50%) of sleep problems and fatigue. The difference across shift systems was significant at *p* < .001 in all cases. Combinations of the most problematic shift characteristics were associated with some increase in negative attitude to the shift schedule. Among schedule characteristics, only long weeks turned out significant in the multivariable regression. The strongest predictor of negative attitude to work hours were social difficulties due to work schedule [ß = 4.98 (95% Confidence interval (Ci) = 3.41, 7.27; *p *< .001], fatigue caused by schedule (ß = 3.20 Ci = 2.03, 5.05; *p *< .001), sleep problems caused by schedule (ß = 2.10 Ci = 1.46, 3.01; *p *= .01), and stressful work (ß = 1.52 Ci = 1.10, 2.11; *p *< .05).

**Conclusion:**

It was concluded that shift systems often included many different shift characteristics, that night shift systems had a large proportion of long shifts, and that split shifts mainly occurred in roster day work. Furthermore, it was concluded that the attitude to the worker’s present shift systems seems to be positive for the majority, with the highest level for those who work either fixed days or nights, compared to those who work alternating shifts (including night shifts). Negative attitude to shift systems was more linked to social difficulties, fatigue or sleep problems due to the shift schedule, than to schedule characteristics per se.

## Introduction

Shift work is associated with impaired health (Kecklund and Axelsson 2016), and particularly night shifts seem important, with disturbed sleep, fatigue, accident risk and cardiovascular disease as outcomes (Sallinen and Kecklund 2010). Other work schedule characteristics that cause sleep difficulties are, for example, short rest between shifts (quick returns) (Sallinen and Kecklund 2010), long shifts (Smith et al. 1998) (Knauth 2007), many consecutive work shifts (Folkard et al. 2005), and direction and speed of shift rotation (von Amelsvoort et al. 2004). In addition, split duty (a long break (> 1.5 h) in the middle of the working day (Anund et al. 2018), on call work (Nicol and Botterill 2004), period planning (regular change of schedule), short notice (before a change of schedule), may be of interest. Regarding the latter two characteristics there seems to be no prior studies available.

One drawback of previous work has been that most original studies have focused on a particular shift system in a particular company/organization, or in a particular occupational group. Thus, local idiosyncratic varieties of shift schedules, work environments, and traditions are likely to have influenced results. In addition, different shift systems (two-shift work, three-shift work, roster schedules (no regular shift cycle) with, or without, nights, permanent night work, etc.), do not seem to have been compared in representative samples from the population. Nor have their components (night shifts, morning shifts, long shifts, short rest, number of successive shifts, etc.), been compared in such samples. Still, there has been attempts to suggest ideal schedules, based on health and safety (Garde et al. 2020).

Furthermore, whereas previous work on problems with shift work has been focused on health (including sleep problems or fatigue) or safety, there is very little information on whether shift workers have a positive or negative attitude to their shift systems. While this topic may have lower importance than health and safety, it is still likely that those who dislike their shift system are likely to seek other employment. Unpopular shift systems or shift characteristics are also less likely to attract new employees.

In a previous study we found that the shift characteristics that constituted a big problem among those who had the characteristic were: short notice (of a new shift schedule), short rest (“quick returns” (< 11 h rest between shifts)), split shifts (having time off > 1.5 h during a shift) long weeks (> 5 successive shifts), and long shifts (> 10 h) (Akerstedt and Kecklund 2017). Night shifts were less often a big problem, as was overtime work. Thus, the results raised questions about the established views on what constituted the most problematic aspects of non-day work, at least from the point of view of the worker.

The previous study had a focus on the separate shift characteristics that constituted a big problem to shift workers. In the present study we focused on the major traditional *shift systems* (three-shift, roster work, etc.) and how they are (1) linked to different types of shift characteristics, particularly those that are (2) seen as big problems, as well as (3) whether workers have a negative or positive attitude to their shift systems. We also focused on whether shift characteristics (4) combine to influence the workers’ attitude to the shift system. Finally, we investigated what occupational groups that dominated different shift systems. To the best of our knowledge, the issues brought up here have never been systematically investigated in a representative sample. For this purpose, we used the originally collected data (Akerstedt and Kecklund 2017).

## Methods

### Participants and data collection

The data collection was carried out by Statistics Sweden, including it in the official, regular Labor force survey (”AKU”). The AKU constitutes a random sample of individuals between 16 and 74 years of age. More than 200,000 individuals from the population register are contacted per year for information on gainful employment and occupation. Questions on background and work force status were added, as well as a question on work hours (any type of non-day work, that is, work outside the interval 0700-1900 h). These questions were asked to half the monthly sample starting in mid-January. When 3500 individuals had responded that they had non-day work, the recruitment of individuals ceased, resulting in 3483 individuals (17 appears to have misunderstood the questions). The number of contacted individuals was 7056, of which 4957 belonged to the labor force, and 462 were unemployed. The potential participants were also asked (via a phone interview) whether they would be willing to fill out a questionnaire on work hours, and questionnaires were sent out to those who accepted participation (two reminders were sent out). The total response rate was 58% (*N* = 2020), but 55 had missing data on shift system, leaving *N* = 1965. The study was approved by the ethical committee of the Stockholm region.

### Measures

The questionnaire was constructed by the authors and contained questions (13) on objective work schedule characteristics. It was initiated with:” Do your work hours at least once per month involve: night work (at least 4 work hours between 2400 and 0600 h), morning work (work shifts that start at 0600 h or earlier), day work (work hours between 0700 and 1900 h), mixed day and night work, shift duration of 10 h or longer, evening work (in the interval 1300 and 0200 h), split duty (more than a 1.5 h break between two parts of the work shift), short daily rest period (< 11 h time off between work shifts), on call work, more than 10 h of overtime work/week (paid or not paid), short notice of the upcoming work schedule (< 1 month ahead), 6 or more shifts in succession, work schedules determined in periods (e.g., a week or month at a time). After each schedule characteristic responded to in the affirmative, the respondent was asked whether it constituted “a big problem in life” (Yes/No).

The respondent was also asked his/her attitude to his/her shift schedule (referring to the *shift system* as a whole, not to single shift characteristics). The responses ranged from 1 = very positive to 5 = very negative. In addition, the respondent was asked about the connection between specific, health related, problems and his/her work schedule. This was formulated as: “Do you feel that your work schedule disturbs your sleep” (yes/no) and was labeled “sleep problems”, “Do you feel that it is difficult to combine your work schedule with seeing family and friends” (yes/no) and labeled “social difficulties”; Do you feel that your work schedule causes fatigue (yes/no) and labeled “fatigue”. Information on gender, marriage status, age, and occupational group were obtained through Statistics Sweden. The respondent was also asked to rate his/her health using a scale, where 1 = very good health, 2 = rather good, 3 = neither good nor poor, 4 = rather poor, and 5 = very poor health. The responses to this question have links to objectively certified illness and all-cause mortality (Ganna and Ingelsson 2015). In addition, two questions asked whether work was physically very heavy (yes/no) or very stressful.

### Statistical analysis

The main analysis was a tabulation of traditional shift systems vs shift characteristics and self-perceived big problems with such shift characteristics, as well as occupational groups. Chi2 analysis was used to compare each shift characteristic across shift systems. The occupational groups were aggregated from occupations in the same area of work. The occupations were obtained from Statistics Sweden, using their official classification system (SSYK).

To understand what combinations of shift characteristics and other variables that resulted in the most negative attitude, a logistic regression analysis was chosen as a tool. The five most problematic shift characteristics, as determined in the previous study (Akerstedt and Kecklund 2017) were used as predictors: Short notice, short rest (< 11 h rest), split duty (> 1.5 h off during a work shift), long weeks (> 5 successive shifts), and long shifts (> 10 h shift duration). All had > 20% prevalence as a big problem among those that had the characteristic. These were entered into a logistic regression analysis, using as outcome the two most negative response alternatives on attitude to one’s work hours (4 + 5, rather negative + very negative). In model 1 each predictor was entered singly. Model 2 included the predictors that were significant in model 1. Interaction terms (products of the variables involved) were computed for variables with significant main effects in model 1. In models 3 and 4 also variables related to physical and mental work, and sleep, fatigue, and social difficulties were added (without interaction analyses).

To obtain an easy to apply indicator of the burden of a shift system based on merely the shift characteristics (night work, long shifts, etc.) of the system, we constructed a “shift load index” (SLI). To do this, we assigned a score of 1 if an individual had one of the five shift characteristics that most individuals saw as a “big problem” in the previous study (Akerstedt and Kecklund 2017), or a score of 2, if the individual had two characteristics, etc. The top five characteristics were short notice, short daily rest, split shifts, long weeks, long shift durations (see “statistical analysis” below). The scores were summed and the scale ranged from 0 to 5. We also analyzed how pairwise combination of shift characteristics were associated with attitude to the shift schedule. The same analysis was applied to combinations of three shift characteristics.

## Results

The sample has been described in a previous publication. However, in brief, 1965 persons were included in the sample, in the age 16–76 years. Of these, 38.2% were males, 48.6% had children and 43.4% were married or cohabiting. 75.9% were in very, or rather, good health (scores 1–2). 32.5% rated their work as physically heavy and 50.1% as stressful. Full-time workers comprised 61.4% of the sample.

### Prevalence of shift characteristics and other variables in shift systems

Table 1 shows that day work was present in all shift systems. Shift systems with night work obviously contained night work, but also day shifts. Roster day work and two-shift work contained evening work and day work (morning work to a lesser degree).

**Table 1:** Shift systems vs prevalence of shift characteristics, and sleep problems, fatigue, and social difficulties, in percent (or *N*) of respondents in shift system

	Day	2-shift	3-shift	Night permanent	Day and night	Roster day	Roster night	Other
*N*	368	679	270	86	179	56	42	285
Women	40.3	32.2	42.3	31.0	**59.5**	21.4	50.0	36.5
Workers	16.9	24.5	**29.0**	21.8	29.0	10.7	28.6	18.7
**Shift timing**								
Day	**97.2**	89.0	93.0	21.0	93.3	92.7	82.9	87.4
Night	7.6	4.0	81.8	**93.0**	83.9	5.4	78.6	28.1
Morning	17.9	28.0	46.1	17.3	36.9	9.6	**56.1**	29.5
Mixed day night	22.4	34.0	87.1	17.3	82.9	20.4	**87.8**	47.6
Evening	63.8	**89.8**	95.9	55.6	76.3	87.0	87.8	86.0
Other shift characteristics								
Split shift	13.4	20.9	13.7	2.5	18.8	**64.7**	24.4	22.3
On call	19.9	7.9	24.5	11.1	45.0	5.8	**53.7**	23.7
< 11 h rest period	23.4	42.2	46.9	14.8	37.6	**66.0**	34.1	39.7
> 10 h shift dur	47.9	35.1	70,3	77.6	86.7	50.0	**90.2**	59.5
≥ 6 successive shifts	30.1	20.3	**37.1**	11.1	32.2	24.1	22.0	30.8
≥ 10 h Overtime	**19.1**	6.5	9.3	12.3	18.0	7.5	2.4	22.0
Period planning	38.0	48.7	59.4	37.0	60.1	**66.7**	51.2	6.6
Short notice	24.7	19.0	21.9	13.6	**33.0**	20.8	26.8	28.4
Sleep, fatigue, social difficulties								
Sleep dist	14.3	36.1	**60.1**	55.3	52.7	34.5	46.3	33.2
Fatigue	31.1	55.9	**66.7**	65.1	61.3	36.4	58.5	45.6
Social difficulties	31.1	52.2	52.3	40.5	50.8	**54.5**	48.8	45.9
Stressful and heavy work								
Stressful work	49.7	55.5	46.9	34.5	54.9	**60.0**	31.0	51.4
Heavy work	28.7	46.4	33.9	44.8	36.3	**51.8**	19.5	38.4

*N* = 1965. All rows show significant differences (*p* =  < .001) after Chi2 analyses. Bold = Highest prevalence in the row

With respect to other shift characteristics, shift systems with night shifts contained much long duration shifts. Among the three-shift workers, short rest periods and long weeks were also prevalent. Roster day work contained much split duty, short rest periods, long shifts, and period planning, while two-shift work contained more modest amounts of the same characteristics. Short notice was moderately present in most shift systems.

With respect to sleep problems, fatigue and social problems due to work hours, the prevalence was high in all non-day work systems, but with a particularly high level in systems with night shifts. Stressful work and heavy work were prevalent in all shift systems, but highest in roster day work and lowest in roster night work.

Sleep problems, fatigue and social difficulties due to work hours had a high prevalence of in all shift systems. The attitude to one’s shift system was most negative for the categories with alternating shift timing, and most positive in day and permanent night work.

### Prevalence of big problems in shift systems

In the shift timing categories, three-shift and roster night work both had a moderate prevalence for night work and mixed day/night work as big problems. Permanent night work showed a low prevalence for those variables as big problems, and a high prevalence (100%) for morning work as a problem. Two-shift and roster night shift systems showed a modest prevalence for evening work as a big problem.

Among other shift characteristics, two- and three-shift systems showed a high or modest prevalence for short rest period, long shifts and long weeks as big problems. Mixed day and night work showed a similar pattern but at somewhat lower levels. Both roster systems had similar prevalence to those of two- and three-shift work, but also very high levels of split duty as a big problem. Roster night also had a high prevalence of big problems with short notice. Permanent night work did not show a big problem for “other characteristics”, except for short notice. The category “other” had a very high prevalence of on call, overtime, and short notice as a big problem.

From the viewpoint of the shift characteristics, split duty was a big problem in the two roster schedules. On call was a big problem in the category “other”. The three categories short rest, long shifts, and long weeks had a moderate to high prevalence for two-shift, three-shift, the two roster schedules, and mixed day/night work. Overtime was a big problem mainly in the category “other”. Short notice was a big problem mainly in roster night work and “other”.

The negative attitude to one’s shift system was most prevalent among those who worked on either three-shift or “roster work with nights” systems and least prevalent among the permanent day and night workers (Table 2). The percent of respondents with a negative attitude was relatively low, however.

**Table 2:** Shift systems vs percentage with big problems among those having a certain characteristic

	Day	2-shift	3-shift	Night -perma-nent	Mixed day/-night	Roster -day	Roster-night	Other	Chi2 or *F*-ratio, *p*
*N*	368	679	270	86	179	56	42	285	
Day	2.2	3.4	2.4	4.3	3.1	2.0	0.0	2.6	2.6
Shift timing									
Night shifts all	2.2	7.8	19.5	2.6	11.4	5.4^#^	**21.2**	12.3	25.1***
Morning shifts	8.1	12.0	11.4	**100.0**	9.1	100.0^#^	8.3	4,6	7.5
mixDN	7.0	19.0	**22.2**	10.0	16.8	18.2^#^	21.1	11.4	15.3*
Evening shifts	7.5	15.8	9.2	4.1	10.7	**19.1**	13.9	8.0	25.3*
Other shift characteristics									
Split shift	13.3	36.4	13.6	10.0^#^	18.9	**51.5**	54.5^#^	21.2	44.0***
On call	9.0	14.3	11.8	11.8^#^	11.6	0.0^#^	18.2	**83.6**	3.3
< 11 h rest period	13.8	**36.1**	34.9	10.5	17.9	22.9	29.4	23.6	30.4**
> 10 h shift dur	19.9	26.1	21.3	3.3	19.5	**31.0**	29.7	17.3	25.1*
≥ 6 successive shifts	22.0	**30.1**	28.6	5.6^#^	25.8	21.4	18.2^#^	13.3	7.4
≥ 10 h overtime	21.6	10.9	8.8	12.3^#^	13.2	0.0^#^	0^#^	**50.4**^**#**^	12.2
Period planning	7.7	10.1	9.0	5.7	9.3	8.1	18.2	**23.2**^#^	4.5
Short notice	27.8	36.4	32.8	33.3	24.6	21.4	46.2	**57.6**	12.6
Attitude and SLI									
Attitude % neg	7.6	16.4	21.2	5.7	10.8	12.5	**24.4**	11.0	40.5***
SLI, mean ± se	1.38 ± .02	1.37 ± .01	1.89 ± .03	1.19 ± .09	2.09 ± .10	2.18 ± .17	1.98 ± .20	1.59 ± .08	*F* = 16.4***

Also, mean ± se for attitude to shift system and shift load index. *N* = 1965. Bold = highest value for row

*SLI* shift load index

*p < .05

**p < .01

***p < .001

^#^*N* < 10 in cell, attitude: 1 = positive—5 = negative

The shift load index had its highest levels among roster day workers, mixed day/night workers, and roster day/night workers. The lowest levels were seen for permanent day work and permanent night work (Table 2).

### Predicting attitude to work hours

To evaluate how combinations of schedule characteristics and other variables would predict attitude to one’s work hours, a logistic regression analysis was carried out with the five highest ranked problem areas (from the previous study) as predictors. The outcome was [“rather negative” plus “very negative” attitude (4 + 5, scored as 1)], versus very positive to [“neither negative, nor positive” (1–3, scored as 0)]. To create a variable that reflected the alternation between night shifts and day, morning, or afternoon or similar shifts, we created a new group that included three-shift work, roster work with night, mixed day and night work and “other” that include night work (*N* = 531), and labeled this “nights&other”. Thus, permanent night work was not included in the analysis, since it was associated with a positive attitude.

Table 3 shows that short rest period, long shifts, long weeks and nights&other were significantly associated with negative attitude when analyzed singly. When the four significant characteristics were entered together in model 2, only long weeks remained significant. No interaction terms could, therefore, be computed. When stress and high physical workload were added in model 3, stress turned out significant, together with long weeks and long shifts. Finally, in model 4, social difficulties, fatigue, sleep problems showed significant ORs, together with stress and long weeks, with social difficulties showing the highest OR.

**Table 3:** Results from hierarchical logistic regression analysis

	Model 1 OR 95% CI	Model 2 OR 95% CI	Model 3 OR 95% CI	Model 4 OR 95% CI
Short rest	1.35 (1.04, 1.76)	1.23 (0.94, 1.61)	1.12 (0.85, 1.48)	0.85 (0.62, 1.14)
Long shifts	1.46 (1.12, 1.90)	1.28 (0.96, 1.71)	1.36 (1.01, 1.82)	1.12 (0.82, 1.54)
Long weeks	1.60 (1.22, 2.11)	1.46 (1.10, 1.95)	1.38 (1.03, 1.85)	1.43 (1.05, 1.94)
Short notice	1.29 (0.95, 1.73)			
Split shifts	1.18 (0.85, 1.62			
Night&other	1.41 (1.06, 1.87)	1.17 (0.86, 1.59)	1.21 (0.89, 1.85)	0.86 (0.61, 1.21)
Stressful work	2.67 (2,01, 3.53)		2.52 (1.88, 3.39)	1.52 (1.10, 2.11)
Physically heavy work	1.34 (1.03, 1.73)		1.07 (0.81, 1.41)	0.89 (0.67, 1.21)
Sleep disturbed by schedule	5.72 (4.30, 7.63)			2.10 (1.46, 3.01)
Fatigue caused by schedule	8.6 0(5.90, 12.24)			3.20 (2.03, 5.05)
Social difficulties due to schedule	9.61 (6.40, 13.10)			4.98 (3.41, 7.27)

Prediction of negative attitude to one’s work hours from shift schedule characteristics, plus stress, physical workload, sleep disturbed by work schedule, fatigue due to work schedule and social difficulties due to work schedule. *N* = 1965. Model 1 = each predictor entered singly. Model 2 = significant shift characteristics from model 1. Model 3 = model 2 plus stress and physically heavy work. Model 4 = Model 3 + disturbed sleep, fatigue, and social difficulties due to work hours. Adjusted for age and gender. “Non-permanent night shifts” refers to all those who have night shifts, but not permanently so

### Shift load and combinations of characteristics

To investigate to what extent combinations of characteristics would be associated with attitude, we carried out an analysis of variance with the shift load index as factor and attitude as outcome, with age and sex as covariates. The ANOVA analysis showed a significant variation (*F* = 4.30, *p *< 0.001) across the six levels (0–5) with a maximum at level 4 (2.48 ± 0.10, *N* = 99) and minimum at 0 (2.16 ± 0.05, *N* = 379). Levels 2–5 had significantly more negative attitude than levels 0 or 1. Thus, the combinations of characteristics led to a more negative attitude compared to no or one characteristic.

Since any of the five most problematic characteristics could contribute to the score, it was of interest also to identify which carried the most weight. Thus, we analyzed all combinations of the five most problematic characteristics, except split duty. The latter had a low co-occurrence with most other characteristics, which caused a sizeable reduction of N. For the pairwise combinations, the analysis of variance showed most negative attitude for long weeks + short notice (2.69 ± 0.22, *N* = 20), followed by long weeks + short rest (2.58 ± 0.15, *N* = 45), and short rest + long shifts (2.39 ± 0.07, 199), with zero combinations = 2.15 ± 0.05, (*N* = 417). All were significantly (*p *< 0.05) different from the group without any combination within the top five problematic characteristics. In the same analysis, also triple combinations were tried, and the triplets with an attitude significantly more negative than zero combinations were: long weeks + short notice, + short rest (2.60 ± 0.20, *N* = 24), and short notice long weeks + short notice + long shifts (2.48 ± 0.14, *N* = 49). All four characteristics reached a negative attitude of 2.44 ± 0.11, *N* = 82, *p *< 0.05 from zero).

### Occupational group characteristic of shift systems

Table 4 shows the prevalence of occupations within shift systems, as well as the prevalence of shift systems in occupations. Day work did not have a dominant occupational group. Two-shift work was dominated by health care assistants and industry/construction workers. Three-shift work was dominated by health care assistants, nurses and MDs, and industry and construction workers. Permanent night work was dominated by health care assistants. Day plus night work was dominated by Health care assistants, transport workers and nurses and MDs. Roster/day work was strongly dominated by health care assistants. Roster/night work was dominated by health care assistants and by transport workers. The category “other” was dominated by health care assistants.

**Table 4:** Shift systems and occupational groups

System → occupation	Day occ/*syst*	2-shift occ/*syst*	3-shift occ/*syst*	Night occ/*syst*	Day + night occ/*syst*	Roster -day occ/*syst*	Roster-night occ/*syst*	Other occ/*syst*
Health care ass								
In occ %	6.7	**42.7**	12.1	7.0	7.0	6.8	2.8	15.0
* In syst %*	*11.1*	*38.5*	*26.5*	*49.4*	*23.2*	***75.0***	*40.5*	*32.3*
Nurses and MDs								
In occ %	9.0	**34.1**	22.3	6.2	14.2	0.9	1.4	11.8
* In syst %*	*5.1*	10.6	*16.8*	*14.9*	***16.2***	*3.6*	*7.1*	*8.8*
Shop assistants								
In occ %	**41.3**	36.4	2.1	0.0	2.1	0.0	0.0	18.2
* In syst %*	*1.2*	***5.8***	*2.4*	*0.5*	*0.8*	*0.1*	*0.1*	*1.8*
Industry and construction work								
In occ %	9.3	**46.6**	19.0	3.6	6.5	0.4	0.4	14.2
* In syst %*	*6.2*	***16.9***	*16.8*	*10.3*	*8.6*	*1.8*	*2.4*	*12.3*
Transport work								
In occ %	12.2	16.3	14.3	3.1	**32.7**	1.0	10.2	10.2
* In syst %*	*3.2*	*2.3*	*5.0*	*3.4*	*17.3*	*1.8*	***23.8***	*3.5*
Restaurant personnel								
In occ %	28.6	**30.6**	16.3	2.0	8.2	2.0	0.0	12.2
* In syst %*	***3.8***	*2.2*	*2.9*	*1.1*	*2.2*	*1.8*	*0.0*	*2.1*
Police, security, fire personnel								
In occ %	13.0	10.1	**30.4**	1.4	24.6	0.0	4.3	15.0
* In syst %*	*2.4*	*1.0*	*7.5*	*1.1*	***9.2***	*0.0*	*7.1*	*3.9*

Bold indicates highest value in the row

Italic values indicate in percent of all in the particular shift system

% *N* = 1965. All analyses across shift systems are significant (CHI2) at *p* =  < .001. In occup = in occupation. In syst = In shift system (Italics)

The analysis of prevalence of shift systems in occupational groups showed that health care assistants mainly had two-shift work, nurses and MDs mainly had two shift work, shop assistants mainly had day work, industry and construction workers mainly had two-shift work, transport workers mainly had day + night work, restaurant personnel did not have any dominating shift system, and police/security/fire fighters mainly had three-shift or day + night schedules.

## Discussion

There are several key results of the present study. One is the observation that all shift timing characteristics were present in all shift systems. Furthermore, alternating night work (including rosters) seems to be a key negative factor, but permanent night work (and day work) seems well tolerated. Problems like social difficulties, poor sleep, and fatigue due to shift schedule, were more closely associated with a negative attitude to shift work than shift schedule characteristics per se*.*

### Prevalence of shift characteristics

In the prevalence analysis of shift timing characteristics in shift systems, most results were expected, since shift systems are mainly classified after the presence of night shift and morning and afternoon shifts. However, it is noteworthy that night work occurred also outside night-oriented shift schedules, and that day work occurred in all shift systems. Possibly, day shifts in night-oriented schedules may be linked to training, or occasional shift changes, but this is speculation.

With respect to the other shift characteristics, it is interesting that split shifts almost exclusively occurred in daytime roster work, that short rest periods had their highest prevalence in roster day work, and that long shifts most frequently occurred in systems with alternating night and other shifts. This type of observations has not been reported before. The reason for the observation on split shifts is probably that work demands in transport work is linked to rush hours, and in home care work to helping the clients to start or end the day. With respect to short rest periods (quick returns), one might speculate, based on anecdotal data, that they are likely to occur to provide a compressed working week or to morning follow-up of work carried out in the evening, such as in health care. There are no available prior data to compare with, however. In addition, long shifts may involve attempts to compress the working week or to reduce the number of shift teams needed. However, also this is speculation based on anecdotal observations.

The presence of disturbed sleep and fatigue due to work schedule was linked to the presence of night shifts, presumably through reduced sleep duration and extended time awake (Garde et al. 2020). However, also, two-shift systems had high levels of fatigue, again probably due to morning shifts curtailing sleep. Social difficulties were relatively high in all non-day work systems, likely due to the difficulties in fitting non-day work schedules to the demands of the day orientation of the surrounding society (Arlinghaus et al. 2019).

Finally, the link between stress and two-shift and roster day work, is likely due to the work situation and both shift systems are dominated by health care assistants, a group that is on a tight schedule providing home care to clients, often with very detailed temporal scheduling.

### Attitudes to shift systems

In general, the percentage with a negative attitude to their work hours was relatively low, less than 25% for the most negative shift system. However, there were clear differences between shift systems. There are no previous systematic studies of this type, but as expected from studies of sleep, fatigue, accident risk, etc. (Kecklund and Axelsson 2016), the most negative attitude was obtained for non-day work in general, in particular for those systems that alternate between night and different types of non-night shifts. Still, night work, in itself, may not be the most serious problem in these systems for workers themselves, since permanent night workers seem to see very little problem in their work schedule, and are as positive to their work hours as day workers. They also appear to see occasional morning shifts as a big problem, which suggests that they belong to the late diurnal type. Interestingly, the permanent night workers still linked their work hours to disturbed sleep, fatigue, and social difficulties, but did apparently not give them much weight in influencing their attitude to their shift system. The observations invite at least two possible explanations. One posits that it is the alternation between night shifts and more day-time oriented shifts that constitute a problem. Another possibility is that permanent night workers constitute a self-selected group of individuals, although we did not collect such information, but the literature suggests this might be the case (Folkard 2008). The two possibilities might, of course, be combined. Selection out of and into shift systems is also a possible explanation of the overall low levels of negative attitudes to shift systems, despite their links to disturbed sleep, fatigue, and social difficulties.

Split shifts, which were common in the roster systems, showed a high prevalence as a big problem in these categories. On call, which was relatively prevalent in the category “other” also showed a very high “big problem” prevalence in that category. The latter category also had a high “big problem” prevalence for overtime and for “short notice”. On call work was prevalent also in day/night and roster night systems. Short rest period was a problem mainly in two-shift work.

With respect to attitude to one’s work schedule, alternating schedules with night shifts were clearly most negative. In addition, the shift load index, which represents an attempted “objective” estimate of the burden of a shift system, was at high levels. However, also two-shift work and roster day work showed a somewhat high prevalence for negative attitude and the latter showed the highest level for the shift load index. Thus, the workers who alternated between different shift types exhibited the most negative attitude to their work hours, regardless of whether they regularly worked nights. There are at least three explanations for the result. First, those who alternate between shift types may have to work shifts they find clearly unsuitable as such for themselves for various reasons (e.g., early mornings, late evenings, nights). Second, it is rather the alternation between shift types in itself than a particular shift type that makes them exhibit a negative attitude. Third, it is a combination of these two factors that underlies the negative attitude. Interestingly our results showed that the workers of the three-shift and “roster-night” systems perceived both night shifts and alternating between day and night shifts as a big problem with a similar prevalence. This finding is in favor of the last-mentioned explanation, which could be the most expected one, given the current research knowledge of multiple pathways and mechanisms, such as circadian disruption, disturbed sleep, and psychosocial stress, underlying the health consequences of shift work (Kecklund and Axelsson 2016).

Night shifts in general are linked to health problems (Garde et al. 2020; Rivera et al. 2020), and even if permanent night work received very positive ratings of attitude in the present study, meta-analyses suggest that there exists an increased risk of, for example, dyslipidemia (Dutheil et al. 2020) and obesity (Sun et al. 2018), compared to rotating (with nights) shift workers. Thus, health problems are still a risk also in permanent night systems. This means that one should probably exercise caution in promoting permanent night work as an alternative to work schedules that include both night and day-oriented work (evenings, days, mornings). In addition, promoting permanent night work would probably make also workers, whose characteristics are not suited for night shifts, to work shift schedules dominated by night work, which, in turn, can be expected to increase the negative health consequences of shift work at the population level.

### Shift load index

The shift load index showed peak load for roster day work, but also for all other systems with night work, except for the lowest point for permanent night work and day work, and on the whole corresponds relatively well to the attitude to the shift system, but not perfectly so. Interestingly, the low shift load index value for permanent night work indicates that the load due to other schedule characteristics is quite low (note that night work is not part of the index). Possibly, this absence of load from other schedule characteristics may contribute to the positive overall attitude to permanent night work. This is speculative, however, and needs confirmation. The intention with the shift load index was to provide a first estimate of whether a schedule should be seen as problematic or not, without collecting data on attitudes, and there seems to be a certain correspondence with attitude. A major discrepancy, however, is the large attitude difference between roster day and roster night schedules. From Table 1 it appears that roster night schedules have a higher prevalence of night work, morning work, on call work, and long shifts. Roster night schedules also have the highest proportion who see night work as a big problem. Possibly, night shifts in roster night work present particular challenges. It is also of interest that such systems have a much larger proportion of transport workers than roster day work. This likely means spending both nights and days away from home, possibly also with poorer sleep arrangements. The importance of spending nights and days away from home should be an interesting question in future studies.

### Predictors of attitude to work hours

Social difficulties, fatigue and disturbed sleep due to work hours added strongly to the negative attitude to work hours. These are likely to be effects of the work schedule with nights and early mornings (Kecklund and Axelsson 2016). To some extent there might also be a presence of insomnia or chronic fatigue states for other than scheduling reasons, although this is not possible to determine in the present material. The strongest predictor was social difficulties, which agrees with present consensus conclusions on social difficulties as an important factor in reactions to shift work (Arlinghaus et al. 2019). However, in the present study we analyzed attitude to the shift schedule using social difficulties, sleep problems, fatigue, stress and shift schedule characteristics simultaneously as predictors. The results suggest that it is not so much the schedule characteristics per se that are associated with the attitude, but rather the individuals’ perception of social difficulties, sleep problems, or fatigue due to the work schedule (and to stress). We suggest that the difficulties with the schedule interact with the individual’s personal resources to handle the schedule. Apparently, a majority of shift workers are able to handle the demands of irregular hours rather well, as indicated in Table 2. Some, obviously, are not. The present study does not contain data for an analysis of factors of tolerance, and previous work is not conclusive (Ritonja et al. 2019). Clearly, there is a need for longitudinal research on this issue.

Stress strongly predicted negative attitude to one’s work hours, but no interaction effect was found. Thus, it seems unlikely that the perception of stress would emanate from a difficult work schedule, and it seems more likely to be due to other job characteristics, such as the demands/intensity of work tasks. The observation that stress was linked to an increase in negative attitude to one’s work schedule may appear strange, but not illogical, since a stressful work situation may intensify workers’ perceptions of the problems they encounter with their work hours. Still, the strength of the influence of stress, compared to that of shift characteristics is remarkable. No similar findings are available in the literature, and there is a need for further work on whether shift work increases the vulnerability to work stress.

The interpretation above raises the question of the extent to which improvements of shift schedules is possible. A minority indicates a negative attitude to their shift schedule, and among those who do, the direct links of to specific shift characteristics are relatively modest. The main problem seems to be the alternation between shifts, particularly when night shifts are regularly involved. The question is, however, whether restrictions of the use of short rest periods, long shifts or long weeks will lead to significant improvements in shift workers’ perception of their work hours. It might even be negative, because the number of days worked would increase and days of recuperation decrease. Permanent night work might be an improvement, if self-selection into night work is feasible overall (which it may not be), with reservations for possible long-term health effects. Thus, it seems that mitigation of negative consequences of shift work (such as sleep and fatigue problems, and social problems) are more important than the design of the shift schedule if the goal is to create attractive working hours from the worker’s point of view. Thus, organizational interventions related to shift scheduling might not be sufficient, and individual-based intervention might be needed to create optimal conditions for shift workers. This should be further explored in well-controlled intervention studies.

### Combinations of shift characteristics

In the analyses of combined effects of different shift characteristics, no significant interactions were found, suggesting that negative characteristics do not potentiate each other. This was somewhat unexpected, but it could be the case that there are built-in compensatory mechanisms. With long shifts, for example 12 h shifts, one also gets fewer days at work, which means longer uninterrupted periods for recuperation, since work hour regulations maximize the number of hours worked per week. Similar effects can result from short daily rest period or long working weeks. In model 2 only long weeks remained significant among the schedule characteristics.

Interestingly, split shifts did not come out significantly in the prediction of attitude. This may be due to its low overall prevalence, but with concentration to mainly roster work. This will have reduced its possibilities to be a strong overall predictor of attitude.

### Occupation and shift systems

The most obvious observation was that roster day work was dominated (75%) by health care assistants. So was permanent night work. Roster night work contained, as discussed above, a large proportion of transport workers. The implications are that estimates of problems in these systems may be partly affected also by the particular job characteristics, including stress and spending days and nights away from home. Thus, care has to be taken that results on health and other issues are not confounded by occupation.

### Implications

The present work has focused on attitudes in relation to shift systems and separate schedule characteristics. While health and safety are of central importance from an occupational health point of view, whether one likes one’s work schedule or not, is likely of major importance to the worker in his/her day-to-day life. It probably also represents some weighted total evaluation of the impact of health and social factors on the individual. It is, probably, this attitude that will influence the willingness to remain in a shift system or to leave. It should, therefore, be a factor of concern for companies that are dependent on a stable labor force. In addition, a labor force with a positive attitude would probably more easily attract new employees. Furthermore, monitoring the attitude to the shift schedule, should give an indication of what needs to be improved to maintain a positive attitude.

### Strengths and limitations

The paper addresses an overlooked, but important, question about workers’ attitude to their own working hours. This is probably a highly relevant topic for companies which want to attract workers and for workers who can decide where to work. Another strength is the representative sample across industries and occupations. Among the limitations are the lack of frequency of exposure to shift characteristics (beyond once a month or more), the cross-sectional design, and absence of data on individual characteristics that might be important for determining shift work tolerance (for example, chronotype, living conditions, family situation, sleep arrangements, and others).

### Conclusion

Traditional shift systems include more shift characteristics than those constituting the core of the systems. Shift systems with night shifts also contained a high proportion of long duration shifts, and split duty was mainly seen in roster day work. The attitude to the one’s shift system was quite positive, with the highest level for those who work either permanent days or nights. Alternation between night and other shifts are associated with a more negative attitude than permanent nights. The attitude to one’s shift system is mainly determined by its social interference, sleep problems, fatigue, and stress, rather than the specific schedule characteristics. Thus, occupational characteristics will be of major importance. It appears that improvements to make a shift schedule attractive should focus on reducing sources of social interference, poor sleep, fatigue and stress. It is important to bear in mind that the attractiveness and healthiness of a shift schedule do not necessarily match.

## Availability of data

Data was collected by Statistics Sweden (the national organization for statistics) and are not available.
